# Vena cava anomalies in thoracic surgery

**DOI:** 10.1186/s13019-018-0704-y

**Published:** 2018-02-01

**Authors:** Andreina Pagini, Massimiliano Bassi, Daniele Diso, Michele Anzidei, Sara Mantovani, Camilla Poggi, Federico Venuta, Marco Anile

**Affiliations:** 1 Division of Thoracic Surgery, Department of General Surgery and Organ Transplant “PARIDE STEFANINI”, Policlinico Umberto I, Sapienza University of Rome, Viale del Policlinico 155, 00161 Rome, Italy; 2 Department of Radiology, Policlinico Umberto I, Sapienza University of Rome, Rome, Italy

**Keywords:** Vascular anomalies, Vena cava, Thoracic surgery, PLSVC, PAPVC, Azygos continuation

## Abstract

**Background:**

Vena cava anomalies are a rare group of anatomical variations due to an incorrect development of the superior or inferior vena cava during fetal life. They generally show no clinical relevance and the diagnosis is done due to the association with congenital heart diseases in most of cases. However, preoperative identification of these anomalies is mandatory for surgeons to proper surgical planning. If not recognized, lethal complications may occur, as already reported in literature.

**Case presentation:**

We report a case series of three different unidentified vena cava anomalies in patients undergoing lung resection. These unrecognized anomalies led to minor complications in two cases and required an accurate intraoperative evaluation in another.

A careful retrospective evaluation of preoperative radiological images showed the anomalies.

**Conclusions:**

A careful evaluation of the vena cava anatomy at pre-operative imaging is mandatory for thoracic surgeons to properly plan the surgery and avoid complications.

## Background

Anomalies of the vena cava represent a rare group of conditions that can occur isolated or in association with other thoracic anomalies, particularly congenital heart diseases (CHD). These conditions are generally asymptomatic and frequently identified as incidental findings during pre-operative computer tomography (CT) or magnetic resonance imaging (MRI). Nevertheless, the knowledge of vena cava anomalies is mandatory for thoracic surgeons and dedicated radiologists to allow safe surgical procedures; if not recognized lethal complications may develop.

We report 3 different cases of vena cava anomalies in patients undergoing lung resection.

## Case presentation

### Case 1. Persistent left superior vena cava

A 73 year-old male with a history of mitral valve regurgitation, treated with biological valve replacement through sternotomy, and previous pacemaker implantation was referred with chest pain and persistent and productive cough. Chest X-ray was performed showing an oval-shaped opacity in the left upper lobe (LUL). Total Body CT scan showed a non-calcific solid nodule of 19 mm, strictly adherent to the parietal pleura in the posterior segment of the LUL. A wedge resection of the LUL was performed through thoracotomy. The intraoperative histological examination of the nodule suggested “pulmonary amyloidosis” and thus the procedure was considered complete.

**Fig. 1:**
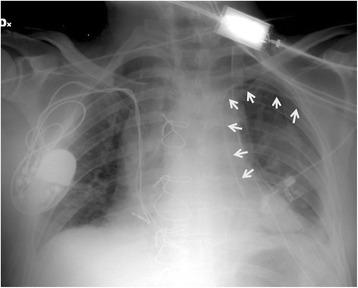
Post-operative X-ray showing the central venous catheter descending on the left side of the mediastinum (arrows)

**Fig. 2:**
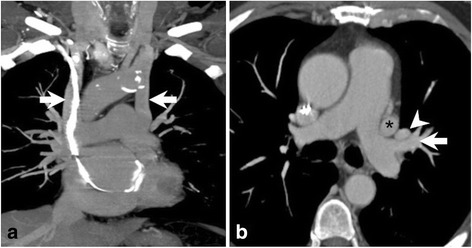
Multiplanar sagittal reconstruction from contrast enhanced CT demonstrate duplication of the superior vena cava (**a**, arrows), with catheters of a pacemaker coursing into the right sided SVC. Axial image demonstrating the relationship between the left sided SVC (**b**, asterisk) and the upper pulmonary artery (**b**, arrow) and upper pulmonary vein (**b**, arrowhead)

During the first post-operative day a chest X-ray was performed showing that the central venous catheter (CVC), placed before the intervention through the left subclavian vein, was located on the left-side of the mediastinum (Fig. 1). Preoperative CT was reviewed and a persistent left superior vena cava (PLSVC) was detected. The anomalous vessel descended laterally to the aortic arch and the left pulmonary artery, anteriorly to the left hilum and finally led to the right atrium through the coronary sinus (Fig. 2). The CVC was removed to avoid complications. The post-operative course was uneventful.

### Case 2. Partial anomalous pulmonary venous connection

A 68 year-old male was referred to the emergency department after repeated syncope episodes. In his medical history the patient reported persistent atrial fibrillation and a previous myocardial infarction treated 6 years ago with percutaneous coronary intervention. CT-scan was performed showing the presence of a solid formation of 10 mm in in the posterior segment of the right upper lobe (RUL). Fine needle ago-biopsy allowed the diagnosis of pulmonary adenocarcinoma and right upper lobectomy was planned.

During the isolation of the upper lobar artery an anomalous vessel located immediately above the lobar artery was injured causing significant bleeding. The vessel was repaired with 5/0 prolene stiches and the bleeding stopped. The injured vessel resulted to be an anomalous branch of the upper pulmonary vein draining into the superior vena cava (SVC). Another analogous vessel was found posteriorly. Both vessels were finally dissected without consequences and surgery was successfully completed.

**Fig. 3:**
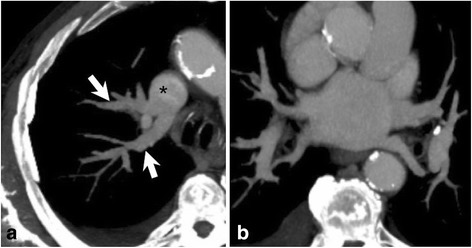
Contrast enhanced CT demonstrate anomalous venous drainage with two pulmonary veins (**a**, arrows) draining the right upper lobe into the superior vena cava (**a**, asterisk). Pulmonary veins are normal (**b**). Findings are consistent with partial anomalous pulmonary venous connection

An accurate examination of preoperative CT images showed the presence of a partial anomalous pulmonary venous connection (PAPVC) between the RUL and the SVC. At the level of the carina, just behind the division of the upper lobar artery, two anomalous vessels drained the apical segments of the RUL into the SVC, while a small superior pulmonary vein drained the other segments into the left atrium (Fig. 3). The postoperative course was uneventful.

### Case 3. Azygos continuation of the inferior vena cava

**Fig. 4:**
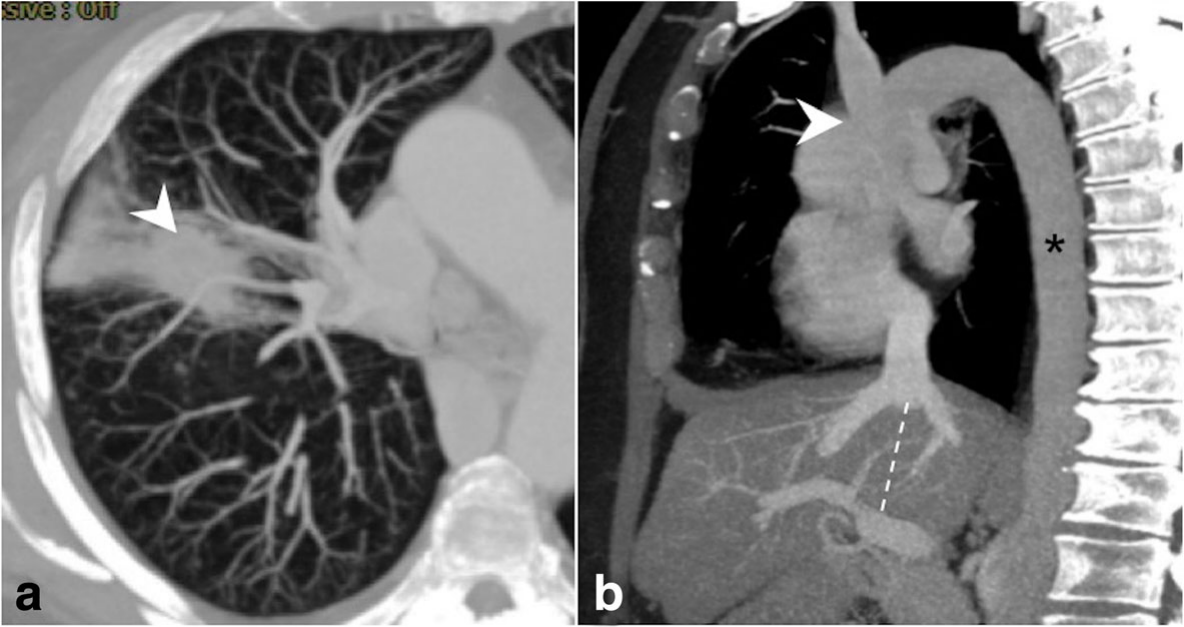
Preoperative CT scan showing right upper lobe squamous carcinoma (**a**, arrowhead). Multiplanar sagittal reconstruction shows enlarged azygos vein (**b**, asterisk) coursing through the diaphragm and draining into the superior vena cava (**b**, arrowhead); the hepatic inferior vena cava cannot be visualized along its normal course (**b**, dashed line). Findings are consistent with agenesis of the hepatic inferior vena cava and azygos continuation

A 62 year-old woman with a history of breast cancer and erythema nodosum came to our department for an incidental finding of a right pulmonary nodule. Preoperative CT scan showed an irregularly-shaped subpleural nodule of 24 × 18 mm in diameter located in the RUL. Another non-calcific nodule of 7 × 6 mm was found in the middle lobe. In addition, CT scan showed the absence of the infrahepatic portion of the vena cava and the presence of an enlarged azygos vein draining all the venous blood from the caudal segments (Fig. 4), known as azygos continuation of the inferior vena cava. Furthermore, three hepatic veins drained the hepatic venous blood into a short inferior vena cava reaching the right atrium.

**Fig. 5:**
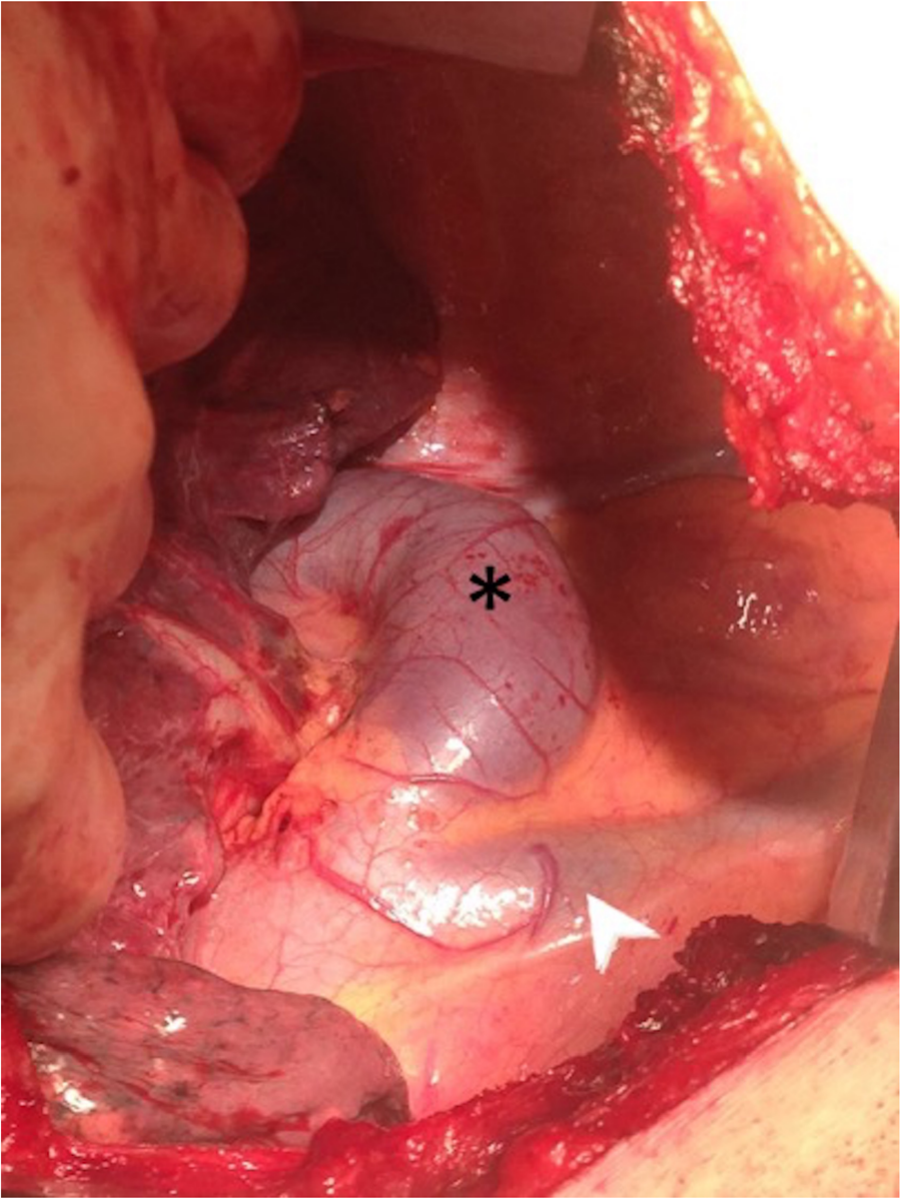
Intra-operative view. The enlarged azygos vein (asterisk) drain into the superior vena cava (arrowhead)

A wedge resection of both nodules was performed. An enlarged azygos vein draining into the SVC was visible beyond the mediastinal pleura (Fig. 5); the dissection during RUL mobilization was carefully performed to avoid damage of the enlarged azygos vein. The histological examination of both nodules showed chronic inflammation with a granulocytic infiltrate. No complications occurred.

## Discussion and conclusions

Although vena cava anomalies are uncommon, their recognition is mandatory for the radiologists and the surgeons and their unacknowledgment could lead to complications during thoracic surgery intervention. Only rarely anomalies of the vena cava are of clinical concern and these are usually diagnosed accidentally or in association with CHD. However, none of our patients had CHD or other vascular anomalies.

PLSVC is the most common anomaly of the thoracic venous system and occurs approximately in 0.5% of the general population [1]. It is due to the failed regression of the left anterior cardinal vein that generally forms the Marshall’s ligament. In 80–90% of cases PLSVC coexists with a right superior vena cava and in up to 65% of these cases a left innominate vein may be completely absent [2]. In approximately 80–90% of cases, the PLSVC drains into the right atrium through the coronary sinus without hemodynamic consequences. Conversely, when it drains into the left atrium, it may result in right-to-left shunt or in hemodynamic overload on the left atrium with the risk of atrial fibrillation or paradoxical embolization. Although it is the most common thoracic venous anomaly, only few cases of lung cancer resection in patients with a PLSVC have been reported ([3, 4]), generally without complications. However, a large PLSVC may disturb mediastinal lymph node dissection or be misdiagnosed as para-aortic nodal metastasis [5]. There is only one report of a T4 lung cancer that involved a PLSVC, treated by Okur et al. with partial resection of the abnormal vessel [6].

PAPVC is a rare congenital defect characterized by the presence of one or more pulmonary veins that drain in the vena cava. It is due to an incorrect development of the fetal pulmonary vein and it is present in 0.4–0.7% of the population. It is often associated with atrial septal defects [7]. PAPVC is generally located in the right lung and the anomalous vessel usually drains into the SVC, as in our patient. However, anomalous connections with inferior vena cava (IVC), right atrium, azygos vein, portal vein, or hepatic veins have been reported in literature [8]. In particular, Cooper first described in 1836 a Scimitar-shaped vein draining part of the right lung into the IVC [9]. The term “scimitar” refers to the resemblance of the anomalous vein to a Turkish sword on chest X-ray. This anomaly is today known as “Scimitar Syndrome”. Anomalous left-sided PAPVC is less common and in this case the anomalous vessels could drain into the left brachiocephalic vein, coronary sinus, or hemiazygos vein. The surgical indication for PAPVC repair is the presence of left-to-right shunt symptoms or a pulmonary-to-systemic flow ratio (Qp/Qs) greater than 2.0 [10]. However, in patients undergoing lung surgery, this anomaly may require preventive treatment in order to avoid hemodynamic complications and even fatal events. Black et al. described a lethal case of right pneumonectomy with left upper lobe anomalous venous connection [11]. In fact, if the PAPVC is in a different lobe of the planned intervention, a major lung resection could cause heart failure due to increased left-to-right shunt. In these cases, hemodynamic assessment before the intervention could be required to identify those cases requiring preventive PAPVC repair [12]. If the PAPVC is located in the resected lobe, as in our case, no hemodynamic changes should appear. In these patients, lobectomy represents an adequate treatment for both lung cancer and PAPVC [13].

Azygos continuation of the inferior vena cava, commonly called simply “azygos continuation”, is a venous anomaly in which an enlarged azygos vein is the direct continuation of the IVC and drains blood from the caudal districts to the SVC. It affects about 0.1–0.6% of the population, with a slight preponderance in males [14]. It is due to the agenesis of the prerenal IVC. In this situation, the venous return is guaranteed by the supracardinal system that normally forms the azygos system. Both azygos and hemizygos continuation can occur, but azygos continuation is much more common [15]. Often, the azygos vein is dilated as reported in our case. Patients with azygos or hemiazygos continuation usually are asymptomatic and do not required any intervention [16]. However, this anomaly can cause complications during surgical and interventional procedures like right heart catheterization, cardiopulmonary surgery and femoral vein catheter advancement. In thoracic surgery, particular care should be paid in case of esophagectomy. In fact, section of the azygos vein could be required during esophagectomy, and it is usually performed without consequences. Nevertheless, in case of azygos continuation it is mandatory to preserve the integrity of the azygos vein given its role in venous return and its dissection could lead to lethal complications. Martín-Malagón reported a case of hypotension, anuria and death after Ivor Lewis esophagectomy in a patient with unacknowledged azygos continuation [17].

In conclusion, in this case-series we report three different vena cava anomalies whose unacknowledgment produced minor complications in two cases. Even if rare, this represents an important group of anatomical variations that could increase the risk of intra-operatory complications during thoracic surgery, and could lead to lethal consequences. Pre-operative evaluation of the vena cava anatomy at CT scan is mandatory for the surgeon to properly plan the surgery. Furthermore, the identification and isolation of mediastinal great vessels should be performed precociously during any thoracic surgery intervention in order to avoid complications.

## Abbreviations

CHD: congenital heart diseases
CT: computer tomography
IVC: inferior vena cava
LUL: left upper lobe
MRI: magnetic resonance imaging
PAPVC: partial anomalous pulmonary venous connection
PLSVC: persistent left superior vena cava
RUL: right upper lobe
SVC: superior vena cava
